# Women’s Perceptions of Cultural Sensitivity of Midwives During Intrapartum Care in Riyadh, Saudi Arabia

**DOI:** 10.3390/healthcare13172172

**Published:** 2025-08-30

**Authors:** Abdulaziz M. Alodhialah, Shorok Hamed Alahmedi

**Affiliations:** 1 Department of Medical Surgical Nursing, College of Nursing, King Saud University, Riyadh 11451, Saudi Arabia; aalodhailah@ksu.edu.sa; 2 Department of Nursing Management and Education, College of Nursing, Princess Nourah bint Abdulrahman University, P.O. Box 84428, Riyadh 11671, Saudi Arabia

**Keywords:** cultural sensitivity, midwifery care, intrapartum care, patient satisfaction, Saudi Arabia

## Abstract

**Background:** Cultural sensitivity during intrapartum care is a critical determinant of maternal satisfaction and quality of care, particularly in multicultural settings. In Saudi Arabia, the diversity of birthing women underscores the need for midwives to provide culturally competent, respectful, and individualized care. **Objective:** To assess women’s perceptions of midwives’ cultural sensitivity during intrapartum care in Riyadh, Saudi Arabia, and identify demographic factors influencing these perceptions. **Methods:** A quantitative cross-sectional study was conducted using a validated cultural sensitivity questionnaire. Data were collected online through purposive sampling from women who had given birth in the past 12 months. Descriptive statistics summarized participant characteristics and perception scores, while inferential tests examined associations between perceptions and demographic variables. **Results:** women reported moderate to high perceptions of cultural sensitivity. Age and nationality significantly influenced perception scores (*p* < 0.05). While communication and respect for religious practices scored highest, areas such as shared decision making and language-concordant support were identified as needing improvement. **Conclusions:** Women in Riyadh often perceive midwives as culturally sensitive; however, gaps remain in communication and involvement in decision making. Training programs that strengthen midwives’ cultural competence—especially in language services and patient engagement—could enhance the intrapartum experience.

## 1. Introduction

Childbirth is a profound life event, shaped not only by clinical care but also by the values, beliefs, and traditions a woman carries with her into the labor ward. Around the world, women approach birth with expectations rooted in their cultural and religious backgrounds, and these expectations often influence how they interpret the quality of care they receive [1]. In Saudi Arabia—where population diversity has grown rapidly over the past decades—this reality is particularly evident. Urban centers such as Riyadh now welcome women from a wide range of ethnicities, languages, and faith traditions, creating a complex tapestry of needs within maternity services [2,3].

In such environments, midwives are positioned at the heart of the birthing experience. Their role extends beyond monitoring contractions or managing delivery; it involves understanding and responding to each woman’s cultural identity. Cultural sensitivity, in this context, means more than avoiding offense—it is about actively incorporating a woman’s beliefs, preferences, and social norms into the care process [4]. This might mean adjusting communication styles, respecting religious rituals, providing privacy that aligns with modesty norms, or offering choices that allow women to feel in control of their birth experience [5,6].

Global health organizations have repeatedly highlighted the importance of this approach. The World Health Organization’s framework for quality intrapartum care includes respectful maternity care as a central pillar, urging providers to recognize and accommodate women’s cultural and personal preferences [7]. Likewise, the International Confederation of Midwives embeds cultural competence within its professional standards, viewing it as an essential skill for safe and respectful practice [8]. In countries like Saudi Arabia—where cultural norms around gender, religion, and modesty play a particularly strong role in healthcare interactions—these principles take on even greater weight [9,10].

Despite this, research in Saudi Arabia has largely concentrated on maternal satisfaction in general terms, with only limited attention to culturally specific elements of midwifery care [11]. International studies have identified critical dimensions of cultural sensitivity—effective communication, language concordance, respect for religious practices, involvement in decision making, and culturally appropriate emotional support—that are associated with better maternal experiences [12]. Yet little is known about how Saudi women, and the many expatriate women who give birth in Saudi facilities, perceive these aspects in practice. Without such knowledge, it is difficult to know where strengths lie and where targeted improvements are needed [13,14].

The context in Riyadh makes this inquiry even more pressing. As the capital city, it attracts a highly diverse population, including a substantial proportion of non-Saudi residents who may face linguistic and cultural barriers when accessing care [15]. For a woman giving birth far from her home country, in a setting where language and customs may differ significantly, the midwife often becomes the key figure who can bridge—or deepen—the gap between expectation and reality [16]. A midwife’s ability to navigate cultural nuances can influence not only the woman’s satisfaction but also her trust in the health system, her willingness to seek care in the future, and even her clinical outcomes [16].

Yet cultural sensitivity is not a fixed attribute. It can be strengthened through education, reflection, and institutional support—or eroded by system pressures, time constraints, and lack of training [17]. In a busy labor ward, where clinical priorities compete with the human need for connection, it is all too easy for cultural considerations to be overlooked. This is why systematic examination of women’s perceptions is so valuable: it provides direct feedback on how well midwives are meeting the challenge and where improvement efforts should focus [18].

The present study was designed with this purpose in mind. It seeks to capture the voices of women who have given birth in Riyadh, asking them to reflect on the cultural sensitivity of the care they received from midwives during labor and delivery. By using a validated survey instrument, the study not only measures overall perceptions but also explores how these perceptions vary by key demographic factors such as age, nationality, and education level. This approach allows us to identify patterns—such as whether expatriate women report lower cultural sensitivity scores than Saudi nationals or whether younger women perceive different strengths and weaknesses compared to older mothers.

### Conceptual Framework

**Figure 1:**
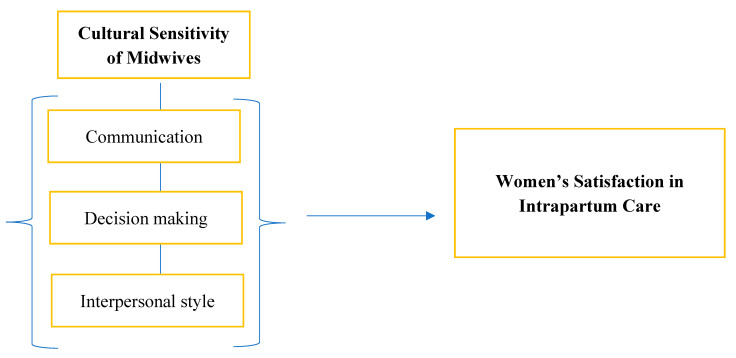
Conceptual framework model (Source: Author’s elaboration).

Figure 1 presents the conceptual framework guiding this study. The independent variables, communication, decision making, and interpersonal style, represent key domains of midwifery care hypothesized to directly influence the dependent variable: women’s satisfaction with intrapartum care. This model supports the examination of how culturally sensitive practices impact overall patient experience.

## 2. Materials and Methods

### 2.1. Study Design

This research adopted a quantitative descriptive cross-sectional design, which is well-suited for capturing a “snapshot” of participants’ perceptions at a single point in time. Such a design allows for the systematic measurement of attitudes, beliefs, or experiences in a defined population without manipulating variables, making it appropriate for exploring how women perceive midwives’ cultural sensitivity during intrapartum care. By using a cross-sectional approach, the study could efficiently collect data from a diverse sample of women in Riyadh, enabling comparisons between subgroups based on demographic characteristics such as age, nationality, and education level.

### 2.2. Study Setting

The study was conducted in Riyadh, the capital city of Saudi Arabia and a major hub for maternal health services in the country. Riyadh is home to a rapidly growing and culturally diverse population, including Saudi nationals and expatriate residents from various regions such as the Middle East, Asia, Africa, and Europe. Maternity care in the city is delivered across a network of public hospitals, private medical centers, and specialized women’s health facilities, all staffed by a mix of Saudi and non-Saudi midwives. This diversity in both patient and provider populations makes Riyadh an ideal setting for examining the role of cultural sensitivity in intrapartum care.

### 2.3. Sample and Sampling Strategy

The study targeted women who had given birth in a healthcare facility in Riyadh within the 12 months preceding data collection. Eligible participants were aged 18 years or older, able to read and understand Arabic or English, and willing to provide informed consent. Women were excluded if they had major cognitive impairments or developmental disabilities that could affect their ability to comprehend survey items, significant language barriers preventing them from completing the survey in Arabic or English, or severe postpartum mental health conditions such as acute psychosis that might compromise recall or perception reporting.

A purposive non-probability sampling technique was employed, as the research sought to include women who could provide firsthand accounts of recent intrapartum care experiences. Recruitment was conducted primarily through online channels, including social media platforms (WhatsApp, Twitter, Instagram) and community-based maternal support groups. The recruitment message contained a brief introduction to the study, eligibility criteria, and a secure link to the online questionnaire. Participants were encouraged to share the survey link with eligible acquaintances (snowballing) to widen reach.

### 2.4. Data Collection Tools

Women’s perceptions were measured using the Cultural Sensitivity in Midwifery Care Questionnaire (CSMCQ), originally developed to assess the extent to which maternity care providers deliver services in a culturally sensitive manner [16]. The tool was designed to capture multiple dimensions of cultural sensitivity during labor and delivery, with the aim of quantifying women’s experiences in a structured and comparable way.

The CSMCQ is composed of 25 items organized into five domains: (1) Communication and Language Support—evaluating clarity, comprehensibility, and language concordance; (2) Respect for Cultural and Religious Practices—assessing accommodation of prayer times, modesty preferences, and religious rituals; (3) Involvement in Decision Making—measuring the extent to which women are included in care decisions; (4) Privacy and Dignity—evaluating measures taken to protect personal privacy; and (5) Emotional and Social Support—capturing interpersonal support during labor. Each item is rated on a 5-point Likert scale ranging from 1 (“strongly disagree”) to 5 (“strongly agree”), with higher scores indicating greater perceived cultural sensitivity. Domain scores are calculated by averaging item responses within each domain, and the overall score is obtained by averaging across all items.

Validity and reliability of the original instrument have been established in prior research, with reported Cronbach’s alpha coefficients exceeding 0.85 for the overall scale and individual domains. For use in this study, the tool underwent a rigorous translation and cultural adaptation process following WHO guidelines for instrument adaptation. Two bilingual healthcare professionals independently translated the questionnaire from English into Arabic, after which an independent bilingual translator, blinded to the original version, performed a back translation into English. An expert panel comprising three midwifery educators, two cultural competence specialists, and two maternal health researchers reviewed both versions to ensure semantic, conceptual, and experiential equivalence. Pilot testing was conducted with 20 postpartum women representing a range of nationalities and education levels to assess clarity and relevance. Minor adjustments were made to wording based on participant feedback. The Arabic version used in this study demonstrated strong internal consistency (Cronbach’s alpha = 0.91) and face validity, confirming its suitability for the Saudi context.

### 2.5. Data Collection Procedure

Data collection was conducted over a 10-week period between March and May 2024. The questionnaire was hosted on a secure online survey platform (Google Forms), accessible via the recruitment link. Upon accessing the survey, participants were first presented with an electronic information sheet describing the purpose of the study, eligibility criteria, voluntary nature of participation, and measures taken to protect confidentiality. Those who agreed to participate provided electronic informed consent before beginning the questionnaire.

The survey took approximately 10–15 min to complete. To prevent duplicate submissions, the platform’s settings were configured to limit one response per device. No personal identifiers such as names or contact details were collected. The survey flow included demographic questions followed by the CSMCQ items. In total, 312 women opened the survey link, 298 provided consent, and 284 completed all questionnaire items (completion rate based on consented participants = 95.3%).

### 2.6. Data Analysis

Survey responses were exported to Microsoft Excel for initial cleaning and then imported into IBM SPSS Statistics version 28 for analysis. Descriptive statistics (means, standard deviations, frequencies, and percentages) were calculated to summarize participant characteristics and CSMCQ scores. Group differences in perception scores were examined using independent-samples *t*-tests (for two-group comparisons) and one-way analysis of variance (ANOVA) with Tukey’s HSD post hoc tests (for comparisons across more than two groups). Pearson’s correlation coefficients were used to assess relationships between continuous demographic variables (e.g., age) and perception scores. The level of statistical significance was set at *p* < 0.05.

### 2.7. Ethical Considerations

The study was reviewed and approved by the Institutional Review Board of [Your University Name] (Approval No. KSU-IRB-2024-116). All participants provided electronic informed consent before data collection. Participation was voluntary, and respondents were free to withdraw at any time before submitting the survey. Data were collected anonymously, stored on password-protected devices, and accessible only to the research team. The study adhered to the ethical principles outlined in the Declaration of Helsinki.

## 3. Results

### 3.1. Participant Characteristics

The study included 187 women who met the inclusion criteria and completed the survey in full. As shown in Table 1, the majority of participants (73.3%) were between 35 and 44 years of age, with only 1.6% younger than 25 years. Most respondents were Saudi nationals (81.8%), and nearly all had attained at least an undergraduate degree (91.4%), reflecting a highly educated sample. Parity patterns revealed that 61.5% had experienced three or more births, indicating considerable exposure to maternity care services. Cesarean delivery was more common than vaginal birth (70.1% vs. 29.9%), suggesting a high prevalence of surgical intervention in this cohort. These characteristics depict a population with extensive birth experience and likely well-developed perceptions of intrapartum care.

### 3.2. Reliability of the Study Instrument

Reliability testing confirmed strong internal consistency across all domains of the Cultural Sensitivity in Midwifery Care Questionnaire. As shown in Table 2, Cronbach’s alpha values ranged from 0.77 for the decision-making domain to 0.95 for interpersonal style, all exceeding the 0.70 threshold for acceptable reliability. This confirms that the instrument was both stable and consistent in measuring perceptions of cultural sensitivity in this population.

### 3.3. Differences in Perceptions by Demographics

Analysis of variance (ANOVA) revealed significant variations in perception scores by age and nationality. As summarized in Table 3, women in the 35–44 year age group reported higher cultural sensitivity scores than their younger or older counterparts (F = 5.12, *p* = 0.002). Saudi nationals also rated midwives’ cultural sensitivity higher than non-Saudis (F = 4.67, *p* = 0.010). No statistically significant differences were found by education level (*p* = 0.065), suggesting that perceptions were less influenced by formal educational attainment.

### 3.4. Descriptive Statistics of Cultural Sensitivity Domains

Overall, perceptions of midwives’ cultural sensitivity were positive, with mean scores above 3.9 in all domains. Table 4 shows that interpersonal style received the highest mean score (M = 4.46, SD = 0.52), indicating strong appreciation for midwives’ respectful and empathetic interactions. Communication (M = 4.21) and emotional support (M = 4.18) also rated highly. Decision making scored lowest (M = 3.92), highlighting a potential gap in shared decision making during labor.

### 3.5. Item-Level Ratings

Closer examination at the item level (Table 5) revealed that “Respect for my cultural beliefs” scored highest (M = 4.54), suggesting this is a core strength of current midwifery practice in Riyadh. Conversely, “I was involved in all decisions about my care” scored lowest (M = 3.89), indicating that women may not always be fully engaged in shared decision-making processes. This reinforces the domain-level findings and signals a clear area for improvement.

### 3.6. Correlation Analysis

Bivariate correlations (Table 6) indicated strong and statistically significant relationships between cultural sensitivity domains and overall satisfaction (all *p* < 0.01). The strongest association was between cultural sensitivity and emotional support (r = 0.93), suggesting that women who felt culturally understood also perceived high levels of emotional support during labor.

### 3.7. Predictors of Satisfaction

Multiple regression analysis (Table 7) identified cultural sensitivity (β = 0.35), responsiveness (β = 0.30), and emotional support (β = 0.28) as the strongest independent predictors of women’s satisfaction with intrapartum care. Decision making, while statistically significant, had the smallest effect (β = 0.15). These findings suggest that enhancing midwives’ ability to deliver culturally sensitive care and respond promptly to concerns could yield the largest gains in maternal satisfaction.

## 4. Discussion

This study provides new insights into women’s perceptions of midwives’ cultural sensitivity during intrapartum care in Riyadh, Saudi Arabia, and identifies demographic factors that influence these perceptions. Overall, participants rated midwives positively across most cultural sensitivity domains, particularly interpersonal style, communication, and respect for cultural beliefs. However, decision-making support emerged as a comparatively weaker area, indicating a need for greater inclusion of women in shared care planning during labor. These findings align with global literature underscoring the importance of cultural competence in fostering trust, satisfaction, and improved clinical outcomes in maternity care [19,20].

### 4.1. Interpretation of Key Findings

The high ratings for interpersonal style suggest that midwives in this study generally demonstrated respectful and empathetic behaviors toward women from diverse backgrounds. Such behaviors are consistent with the principles of respectful maternity care advocated by the World Health Organization, which emphasize kindness, active listening, and individualized attention [21,22]. These qualities have been shown to mitigate childbirth-related stress and promote a sense of dignity for women during labor [23].

Communication also scored highly, reflecting midwives’ ability to convey information clearly and maintain open dialogue with laboring women. In culturally diverse settings such as Riyadh, effective communication often requires adaptation to linguistic needs and sensitivity to nonverbal cues [24]. The high scores in this domain suggest that many midwives are adept at bridging potential cultural and language barriers, perhaps aided by bilingual skills or use of interpreters. Nevertheless, disparities in ratings between Saudi and non-Saudi participants indicate that not all women benefit equally, echoing studies from similar multicultural contexts where language discordance remained a persistent challenge [25].

Decision making, although positively rated overall, lagged behind other domains. Shared decision making is increasingly recognized as a critical component of high-quality maternity care, yet its practice can be constrained by hierarchical care structures, time pressures, and cultural norms that may discourage women from voicing preferences [26,27]. In Saudi Arabia, decision making during labor may also be influenced by family involvement and institutional protocols, which can shift agency away from the birthing woman. The lower scores here align with findings from other Middle Eastern studies where women reported limited participation in key clinical decisions [28].

### 4.2. Comparisons with Existing Literature

Our results corroborate previous Saudi research that highlighted strong provider–patient rapport but noted gaps in autonomy and decision making [29,30]. In a multi-country survey of Arab women, interpersonal respect was consistently high, yet opportunities for informed choice and consent varied widely by setting [31]. Similar patterns have been observed in Western contexts, where interpersonal warmth does not always translate into full partnership in care [32]. This suggests that, while relational and emotional aspects of care may be prioritized, structural and systemic changes are needed to embed shared decision-making into routine practice.

The finding that older women (35–44 years) and Saudi nationals reported higher cultural sensitivity scores merits consideration. Older women may have greater familiarity with local maternity care systems and thus feel more comfortable navigating them, or they may hold expectations more closely aligned with prevailing care models. In contrast, expatriate women may encounter unfamiliar protocols, language barriers, or limited accommodation of cultural rituals, which could lower their perception of sensitivity. These dynamics are consistent with reports from other multicultural maternity settings, where migrant women often face higher risks of miscommunication and dissatisfaction [33].

### 4.3. Implications for Practice

The findings underscore the need for targeted interventions to strengthen decision-making support and ensure equitable cultural sensitivity for all women, regardless of nationality. Training programs for midwives should include advanced communication strategies for shared decision making, emphasizing the importance of eliciting women’s preferences and providing balanced, comprehensible information about options during labor. Incorporating simulation-based training that features diverse cultural scenarios could enhance midwives’ readiness to manage complex cultural interactions.

Healthcare facilities should also consider institutional policies that promote autonomy, such as structured consent processes for common interventions and the routine offering of birth plans. For expatriate women, providing language-concordant care—through hiring multilingual staff or expanding interpreter services—could improve both understanding and comfort.

### 4.4. Policy and System-Level Considerations

At the policy level, integrating cultural competence into midwifery education curricula and national clinical guidelines would standardize expectations for culturally sensitive care. Aligning with international frameworks such as the International Confederation of Midwives’ Essential Competencies could help ensure that Saudi midwives meet globally recognized benchmarks for respectful and individualized care [25]. Monitoring and evaluation mechanisms, including patient-reported experience measures, could be embedded into routine quality assurance to track progress over time.

### 4.5. Strengths and Limitations in Context

A key strength of this study is its focus on women’s direct perspectives, gathered through a validated and culturally adapted instrument with strong internal consistency. The inclusion of both Saudi and non-Saudi women enhances the relevance of findings for diverse patient groups. However, the purposive sampling strategy and online recruitment limit the generalizability of results, as participants were likely more educated and technologically literate than the general population. The inability to calculate a traditional response rate, inherent to open online surveys, further constrains external validity. Additionally, self-reported perceptions are subjective and may be influenced by recall bias or social desirability.

### 4.6. Future Research Directions

Future research should adopt mixed-methods approaches to complement quantitative ratings with qualitative narratives that explore the nuances of cultural sensitivity in greater depth. Longitudinal studies could examine whether targeted training interventions lead to measurable improvements in women’s perceptions over time. Comparative research across different regions of Saudi Arabia would also be valuable, given potential variations in cultural demographics and healthcare infrastructure.

## 5. Limitations of the Study

Several limitations should be acknowledged when interpreting these findings. First, the use of a purposive, non-probability sampling strategy and online recruitment via social media may have introduced selection bias, favoring women who are more educated, technologically literate, and engaged with online platforms. This limits the generalizability of the results to the wider population of childbearing women in Riyadh or other regions of Saudi Arabia. Second, as the survey was open to voluntary participation without a defined sampling frame, a traditional response rate could not be calculated, and the degree of non-response bias remains unknown. Third, data were based on self-reported perceptions, which are inherently subjective and may be influenced by recall bias or social desirability. Fourth, the cross-sectional design captures perceptions at a single point in time and cannot establish causality between midwives’ behaviors and women’s satisfaction. Lastly, while the survey instrument underwent rigorous translation and cultural adaptation, it relied on structured items and did not capture the full depth and complexity of women’s experiences, which could be explored more comprehensively through qualitative methods.

## 6. Conclusions

This study highlights that women in Riyadh generally perceive midwives’ intrapartum care as culturally sensitive, with interpersonal style, communication, and respect for cultural beliefs rated most highly. However, decision-making support emerged as the least favorable domain, pointing to the need for greater involvement of women in care planning and clinical decisions during labor. Differences in perceptions between Saudi and non-Saudi women indicate that language barriers, cultural unfamiliarity, and institutional practices may contribute to unequal care experiences. Addressing these gaps through targeted professional training, enhanced interpreter and language-concordant services, and policies that embed shared decision making into maternity care protocols can help ensure more equitable, respectful, and individualized care for all women, regardless of cultural background.

## Figures and Tables

**Table 1:** Demographic characteristics of participants (N = 187).

Variable	n	%
Age		
<25 years	3	1.6
25–34 years	22	11.8
35–44 years	137	73.3
≥45 years	25	13.4
**Nationality**		
Saudi	153	81.8
Non-Saudi	34	18.2
**Education**		
High school diploma	16	8.6
Undergraduate	121	64.7
Postgraduate	50	26.7
**Number of births**		
First birth	31	16.6
Second birth	41	21.9
Third or more	115	61.5
**Type of birth**		
Vaginal	56	29.9
Cesarean	131	70.1

**Table 2:** Reliability analysis of cultural sensitivity domains.

Domain	Items	Cronbach’s Alpha
Communication	5	0.87
Decision making	3	0.77
Interpersonal style	11	0.95

**Table 3:** ANOVA results for perceptions of cultural sensitivity by demographics.

Variable	F	*p*-Value
Age	5.12	0.002 **
Nationality	4.67	0.010 *
Education	2.89	0.065

* *p* < 0.05, ** *p* < 0.01.

**Table 4:** Descriptive statistics for cultural sensitivity dimensions.

Domain	Mean	SD	Min	Max
Communication	4.21	0.64	3.04	4.97
Decision making	3.92	0.71	2.53	4.98
Interpersonal style	4.46	0.52	3.62	5.00
Emotional support	4.18	0.58	3.12	4.98

**Table 5:** Selected item-level means and SDs.

Item	Mean	SD
Communication clarity	4.22	0.61
Responsiveness to concerns	4.07	0.60
Respect for cultural beliefs	4.54	0.56
Emotional support provided	4.18	0.58
Involvement in decision making	3.89	0.73

**Table 6:** Correlations between cultural sensitivity domains and satisfaction (N = 187).

Domain	Communication	Responsiveness	Cultural Sensitivity	Emotional Support	Decision making	Overall Satisfaction
Communication	1	0.85 **	0.88 **	0.87 **	0.74 **	0.79 **
Responsiveness	0.85 **	1	0.92 **	0.89 **	0.77 **	0.81 **
Cultural Sensitivity	0.88 **	0.92 **	1	0.93 **	0.78 **	0.83 **
Emotional Support	0.87 **	0.89 **	0.93 **	1	0.75 **	0.82 **
Decision making	0.74 **	0.77 **	0.78 **	0.75 **	1	0.75 **
Overall Satisfaction	0.79 **	0.81 **	0.83 **	0.82 **	0.75 **	1

** *p* < 0.01.

**Table 7:** Multiple regression predicting satisfaction with intrapartum care.

Predictor	β	t	*p*-Value
Communication	0.25	4.21	0.001 **
Responsiveness	0.30	4.75	0.000 **
Cultural sensitivity	0.35	5.50	0.000 **
Emotional support	0.28	4.40	0.000 **
Decision making	0.15	2.50	0.012 *

* *p* < 0.05, ** *p* < 0.01.

## Data Availability

All data is available in the manuscript.
